# Intrinsic Hippocampal Excitability Changes of Opposite Signs and Different Origins in CA1 and CA3 Pyramidal Neurons Underlie Aging-Related Cognitive Deficits

**DOI:** 10.3389/fnsys.2016.00052

**Published:** 2016-06-09

**Authors:** M. Matthew Oh, Dina Simkin, John F. Disterhoft

**Affiliations:** Department of Physiology, Feinberg School of Medicine, Northwestern UniversityChicago, IL, USA

**Keywords:** postburst afterhyperpolarization, Kv4.2/4.3 channels, calcium, calcium buffer capacity, calcium hypothesis of brain aging

## Abstract

Aging-related cognitive deficits have been attributed to dysfunction of neurons due to failures at synaptic or intrinsic loci, or both. Given the importance of the hippocampus for successful encoding of memory and that the main output of the hippocampus is via the CA1 pyramidal neurons, much of the research has been focused on identifying the aging-related changes of these CA1 pyramidal neurons. We and others have discovered that the postburst afterhyperpolarization (AHP) following a train of action potentials is greatly enlarged in CA1 pyramidal neurons of aged animals. This enlarged postburst AHP is a significant factor in reducing the intrinsic excitability of these neurons, and thus limiting their activity in the neural network during learning. Based on these data, it has largely been thought that aging-related cognitive deficits are attributable to reduced activity of pyramidal neurons. However, recent *in vivo* and *ex vivo* studies provide compelling evidence that aging-related deficits could also be due to a converse change in CA3 pyramidal neurons, which show increased activity with aging. In this review, we will incorporate these recent findings and posit that an interdependent dynamic dysfunctional change occurs within the hippocampal network, largely due to altered intrinsic excitability in CA1 and CA3 hippocampal pyramidal neurons, which ultimately leads to the aging-related cognitive deficits.

## Introduction

The advent of better health care has led to longer lifespan, and thus an increase in the aging population. Concomitant with this longevity, the prevalence of aging-related neurodegenerative diseases (e.g., Alzheimer’s, Parkinson’s, etc.) and their associated neurocognitive deficits is also on the rise. The “normal” aging population is not spared from cognitive deficits, as many without a neurological disorder will also suffer from learning and memory impairments. For example, those diagnosed in the mild cognitive impairment category may or may not progress to Alzheimer’s disease (AD). Hence it is urgent to identify the mechanisms that underlie the normal aging-related cognitive deficits, in addition to those that lead to the neurodegenerative disorders, to prevent a catastrophic strain on the healthcare system and also to better the quality of life as we age.

For that purpose, the hippocampus and its associated medial temporal lobe structures have received the majority of scientific attention, mostly due to case studies of amnesic patients: most well-studied and known being Henry Gustav Molaison (1926–2008). These human case studies and subsequent animal experiments strongly implicated the proper function of hippocampal circuitry as being essential for formation of personal (or declarative) memories, but not motor (or procedural) learning (Dickerson and Eichenbaum, 2010; Squire and Wixted, 2011; Oh and Disterhoft, 2014). Specifically, the CA1 region of the hippocampus received much attention with the revelation that anterograde amnesia can be caused by a complete bilateral lesion limited to CA1 area of the hippocampus as observed in patient RB and others (Zola-Morgan et al., 1986; Rempel-Clower et al., 1996). Hence much focus has been on the CA1 region of the hippocampus to identify the maladaptive cellular and neural network changes that occur with normal aging.

We, too, have focused our attention on the CA1 region to identify the cellular changes that occur within the principal neurons with normal aging. Based upon our observations, we have hypothesized that the major impediment to successful learning is the enlarged postburst afterhyperpolarization (AHP) in aged CA1 pyramidal neurons, which limits the firing of these neurons and thus their recruitment into the neural circuitry underlying memory formation. Furthermore, we suggested that normal aging-related cognitive deficits could be overcome by restoring the postburst AHP of the aged neurons to a young-like state (Disterhoft and Oh, 2006, 2007). However, it has recently been discovered that the necessary changes in one subregion may not be a cure-all for the aging-related changes throughout the brain, such as the CA3 hippocampal pyramidal neurons (Simkin et al., 2015). In addition to the latter, we were able to directly test a key assertion made in the “calcium hypothesis of brain aging” (Khachaturian, 1989, 1994) by using calcium imaging techniques and a multiphoton laser scanning microscope. Hence we will discuss our recent findings and posit our amended hypothesis in this review.

## Aging-Related Changes in CA1

The “calcium hypothesis of brain aging” was proposed in the early 1980’s (reviewed and revised in: Khachaturian, 1989, 1994) based on the published information available at the time; e.g., increases in Ca^2+^-dependent postburst AHP in CA1 pyramidal neurons in hippocampal slices (Landfield and Pitler, 1984; Moyer et al., 1992) and in Ca^2+^ accumulation in synaptosomes prepared using whole brain tissue (Michaelis et al., 1992) from aged rats. In essence, the hypothesis states that the “cellular mechanisms that regulate the homeostasis of cytosolic free Ca^2+^ (i.e., Ca^2+^ ion channel number and/or function, in Ca^2+^ binding proteins, and in Ca^2+^ buffer capacity) are changed with normal aging. Importantly, the direction of the change(s) was not specified. Regardless, the general consensus in the aging field was that the resting levels of free Ca^2+^ ions in CA1 pyramidal neurons are elevated based on reports using bulk loaded fluorescent calcium indicators in dissociated neuronal, hippocampal slice, or cultured neuronal preparations (Kirischuk and Verkhratsky, 1996; Tonkikh et al., 2006; Hajieva et al., 2009) although there are reports that no change in resting [Ca^2+^] is observed with aging in CA1 neurons from hippocampal slices (Thibault et al., 2001; Gant et al., 2006). Furthermore, without experimental evidence from CA1 pyramidal neurons, it is generally presumed that the Ca^2+^ buffering capacity in these neurons is reduced with aging (Tonkikh et al., 2006; Kumar et al., 2009); although increased calcium buffer capacity has been reported in dissociated basal forebrain neurons of aged animals (Murchison and Griffith, 1998). Hence, after nearly 30 years of research, those neuroscientists studying normal aging and aging-related neurological disorders, such as AD, were still unsure as to how resting [Ca^2+^] and its buffering capacity are altered by normal aging in hippocampal pyramidal neurons.

**Figure 1:**
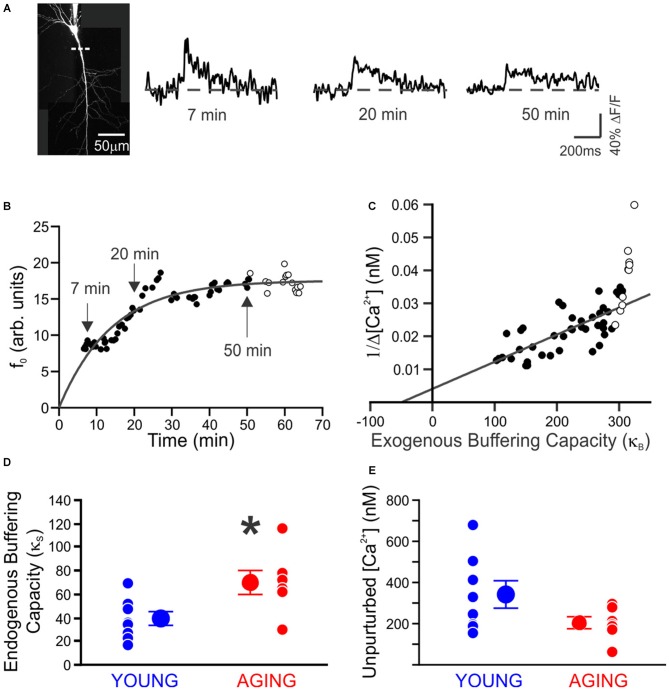
**Endogenous buffer capacity and unperturbed calcium transient measured in individual neurons. (A)** Maximum intensity projection of a neuron. Measurements were made using a line scan (dashed line) 40–80 μm from the soma. Example calcium transients taken during loading of 100 μM OGB-1, 7, 20, and 50 min after breaking whole-cell. **(B)** Example of the increase in resting fluorescence (f_0_, arbitrary units) over time as OGB-1 diffused into and approached a steady-state concentration in the dendrite. Points are fit with an exponential. **(C)** Example of endogenous buffer capacity calculated by back-extrapolation of the relationship between the reciprocal amplitude of calcium transients (1/Δ[Ca^2+^]) plot against exogenous buffer capacity (κ_B_) for the results in **(B)**. In this example neuron, there was an abrupt reduction in the amplitude of Ca^2+^ transients at 50 min, causing an upswing in the plot. The points after 50 min (open circles) were discarded before fitting a line through the results. **(D)** The endogenous buffer capacity is significantly enhanced in neurons from aging rats (*p < 0.05, unpaired *t* test). **(E)** Peak amplitude of the unperturbed calcium transient evoked with a single action potential is not significantly altered with aging (young, 8 neurons from 4 rats, 342 ± 65 nM; aging, 7 neurons from 5 rats, 206 ± 29 nM). Reprinted with permission from Oh et al. (2013) © by the Society for Neuroscience.

**Figure 2:**
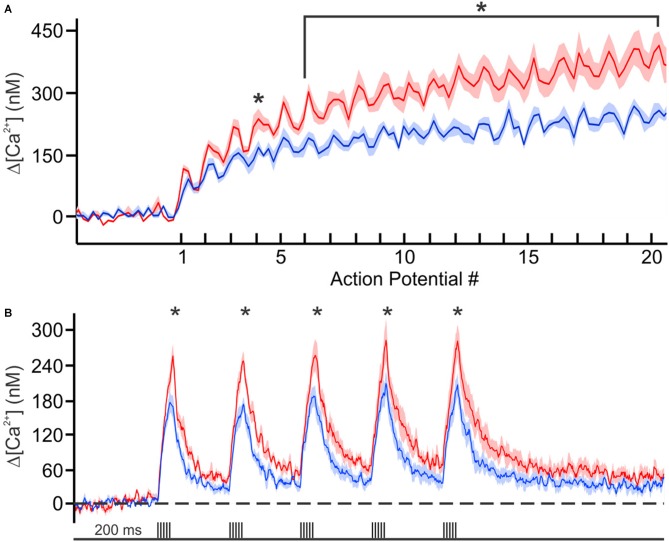
**Calcium concentration during trains of action potentials. (A)** Mean calcium concentrations (shaded regions represent SEM) during 100 Hz trains in young (blue, *n* = 6 from 2 rats) and aging (red, *n* = 6 from 3 rats) neurons loaded with 150 μM OGB-6F. **(B)** Calcium concentration during theta burst activity (5 action potentials at 100 Hz) at theta frequency (5 Hz). Significant differences were revealed with repeated analysis of variances (ANOVAs) for the 100 Hz and theta burst activity. Further analysis revealed significant differences between the age groups at various points during the train of activity. *p < 0.05. Reprinted with permission from Oh et al. (2013) © by the Society for Neuroscience.

Recently we decided to address this void in knowledge by using Ca^2+^-imaging techniques to directly compare the resting [Ca^2+^] and its buffering capacity of CA1 pyramidal neurons from young adult and aged rats (Oh et al., 2013). Contrary to the generally held belief, we found that the resting [Ca^2+^] was reduced while the endogenous Ca^2+^ buffer capacity was increased in CA1 neurons from aged animals (Figure 1). Furthermore, the increased buffer capacity in aged CA1 neurons was able to keep the action potential evoked rise in cytosolic calcium levels in check, to comparable levels observed in young adult neurons. However, during a train of action potentials, the rise in calcium level overwhelmed the increased endogenous buffer capacity and resulted in a much higher levels of calcium in aged CA1 neurons (Figure 2). Notably, the size of the postburst AHP is dependent on the number of action potentials within the burst: more action potentials, the larger the postburst AHP (Landfield and Pitler, 1984; Wu et al., 2004). Based on these findings, we hypothesize that calcium buffer capacity may be increasing during normal aging in CA1 pyramidal neurons to counteract the increase in activity-evoked cytosolic calcium levels; perhaps due to increased influx through voltage-gated calcium channels (VGCC) and/or calcium-induced calcium release (CICR). This increased buffer capacity is limited to allow the cells to adapt to the rise in calcium levels with a couple of action potentials, but not to a train of action potentials. Thus, further increasing the calcium buffer capacity of aged CA1 neurons may be beneficial in restoring the intrinsic excitability of aged CA1 neurons to a young-like state and ameliorate the cognitive deficits in aged subjects. However, the source of the increased calcium buffer capacity is yet to be determined. There are >200 proteins that can bind calcium (Heizmann, 1999; Schwaller, 2009), yet only a few have been carefully examined. Of those, calbindin-D28k and hippocalcin are reduced in CA1 region of aged animals (de Jong et al., 1996; Furuta et al., 1999). Hence, identifying the source of enhanced calcium buffering in the hippocampus may lead to the development of therapeutic strategies to rescue age-related cognitive deficits.

These recent Ca^2+^-imaging findings further support the hypothesis that the enlarged postburst AHP in aged CA1 neurons is due in part to the increased cytosolic calcium during the burst of action potentials. However, it remains to be determined if the increased calcium level during the burst is due to enhanced calcium influx via VGCCs and/or to increased CICR from internal calcium stores (such as the endoplasmic reticulum).

The L-type VGCCs have been shown to be a major source of calcium influx in CA1 pyramidal neurons. In the early 2000’s, western blot and immunohistochemical methods revealed that there may be an increased expression of Cav1.3 subunit of L-type VGCC in the CA1 region of aged rats (Veng and Browning, 2002). This molecular evidence fit well with electrophysiological data that showed increased calcium influx via L-type VGCCs in aged CA1 pyramidal neurons (Thibault and Landfield, 1996; Thibault et al., 2001; Power et al., 2002) which also resulted in larger postburst AHP in these neurons (Moyer et al., 1992; Norris et al., 1998). Hence, it was hypothesized that the amelioration of aging-related learning deficit with systemic administration of nimodipine, an L-type VGCC blocker, was due in part to reduced postburst AHP in CA1 pyramidal neurons which led to increased basal firing of these neurons *in vivo* during learning (Deyo et al., 1989; Thompson et al., 1990; Moyer et al., 1992). However, the postburst AHP is still evident after pharmacological block of L-type VGCCs (Moyer et al., 1992; Power et al., 2002) and in CA1 pyramidal neurons from L-type VGCC knockout mice (Gamelli et al., 2011). Furthermore, recent molecular examination of dorsal hippocampus revealed a reduction in total, but an increased surface ratio, of Cav1.2 L-type VGCC expression levels in dorsal CA1 region of aged rats as compared to young adult rats (Núñez-Santana et al., 2013). These latter findings suggest that changes in function, rather than absolute number, of L-type VGCCs underlie the increased calcium influx into CA1 pyramidal neurons in aged animals.

CICR is another major source of calcium for the postburst AHP (Torres et al., 1996) that is elevated in CA1 pyramidal neurons in aged animals (Kumar and Foster, 2004; Gant et al., 2006). Recently, two cellular mechanisms that underlie the increased CICR in aged CA1 pyramidal neurons have been proposed. One proposed mechanism suggests that increased oxidative stress on ryanodine receptors is the cause for the enhanced CICR, as reducing the oxidative state of ryanodine receptors with dithiothreitol (DTT) reduced the postburst AHP only in CA1 pyramidal neurons from aged animals (Bodhinathan et al., 2010; Foster, 2012). The second proposed mechanism is that the level of an immunophilin, which normally inhibits calcium release by binding to and stabilizing the ryanodine receptors in a closed state (FK506-binding protein 12.6/1b: FKBP1b) is reduced in CA1 pyramidal neurons in aged animals (Gant et al., 2011, 2015). Notably, when the FKBP1b expression was reduced in young adult rats, the postburst AHP was enlarged in CA1 pyramidal neurons (Gant et al., 2011); whereas, when the FKBP1b expression was enhanced in aged rats, the postburst AHP was reduced in CA1 neurons (Gant et al., 2015). Regardless of the mechanism, reducing CICR’s contribution to the rise of cytosolic levels of calcium may be beneficial in reducing the postburst AHP in aged CA1 neurons, which in turn may ameliorate the cognitive deficits in aged subjects.

The results from nearly 40 years of examining aging-related alterations in CA1 pyramidal neurons has led to many generalizations that have been hypothesized to occur in other brain regions. However, as we recently discovered, mechanisms that underlie aging-related changes in one brain region are not necessarily the same in other brain regions, even those in close proximity to CA1.

## Aging-Related Changes in CA3

Until recently, aging-related changes in CA3 pyramidal neuron activity have been examined at a systems level by using *in vivo* unit recordings and functional magnetic resonance imaging (fMRI) blood oxygen level-dependent (BOLD) measurements. A series of *in vivo* place field recording studies demonstrated the inflexibility of place fields, due to higher firing rates, of CA3 neurons in aged learning-impaired rats (Wilson et al., 2003, 2005). These place field studies were complemented by fMRI studies that demonstrated increased BOLD activity in the CA3/dentate gyrus (DG) region in aged human subjects who were impaired on pattern separation tasks (Yassa et al., 2011) and in early AD and mild-cognitive impaired patients (Dickerson et al., 2005; Bakker et al., 2012). While these studies strongly implicate increased firing rate in CA3 region as a source of aging-related cognitive deficits, the cellular mechanism(s) that underlies the aberrant firing rate was yet to be determined.

**Figure 3:**
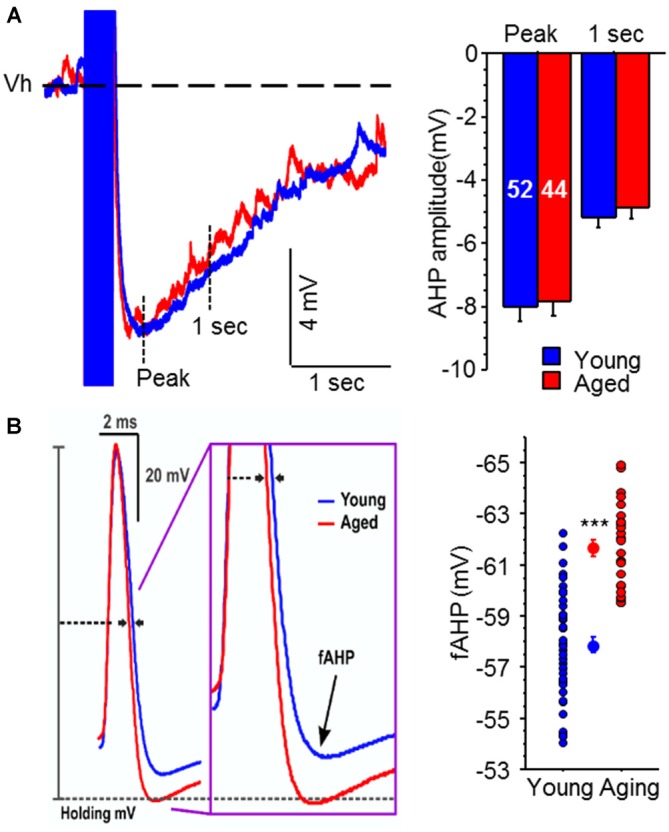
**Medium and slow postburst afterhyperpolarizations (AHPs) are not altered, but the fast AHP is increased, with aging in CA3 pyramidal neurons. (A)** Illustrated are examples of postburst AHPs evoked with a 50 Hz train of suprathreshold current injections into young (blue) and aged (red) CA3 pyramidal neurons. APs are truncated for clarity. All AP measurements were performed with the neuron held at near −60 mV (Vh: dotted line). No significant age-related difference was observed in either the medium or slow postburst AHPs, measured at the peak and 1 s interval after last current pulse, respectively. The number of cells (*n*) is represented in the middle of each bar for each group. **(B)** The fAHP evoked with a single orthodromic stimulus was significantly enhanced (more hyperpolarized) in aged CA3 neurons than young neurons. Illustrated are overlays of representative traces from young (blue; *n* = 43) and aged (red; *n* = 28) CA3 neurons that show fAHP evoked with single orthodromic AP. fAHP is measured as absolute membrane voltage. Error bars are SEM. ***p < 0.0005 (Fisher’s PLSD *t* test). Modified and reprinted with permission from Simkin et al. (2015) © by the Society for Neuroscience.

Recently we decided to tackle this issue by conducting a systematic evaluation of age-related alterations in intrinsic membrane properties of CA3 pyramidal neurons from young adult and aged rats (Simkin et al., 2015). Surprisingly and counter to expectations based on evidence from CA1 pyramidal neurons, the postburst AHP was similar in CA3 pyramidal neurons from both young adult and aged rats (Figure 3). However, the fast AHP, which is not altered with age in CA1 pyramidal neurons (Matthews et al., 2009), was significantly enhanced in CA3 pyramidal neurons from aged rats due to increased expression of Kv4.2/4.3 potassium channel expression in the perisomatic area of CA3 region (Simkin et al., 2015). Functionally, the enhanced fast AHP could reset the ionic mechanisms (e.g., voltage-gated sodium channels) necessary to generate an action potential quicker and thus allow another action potential to be generated in rapid succession: i.e., allow faster firing rate. This latter perspective fits with the *in vivo* place field recording studies that found increased firing rate in CA3 neurons (Wilson et al., 2005), which in turn could lead to increased recurring activity of the CA3 network via its auto-associative, recurrent collaterals. This increased CA3 network activity could lead to the overgeneralization observed in aged subjects, which lead to deficits in separating experiences or memories with similar features (Wilson et al., 2005; Haberman et al., 2011; Yassa et al., 2011; Bakker et al., 2012). Although these recent fast AHP changes were observed in behaviorally naïve animals, we found that blocking Kv4.2/4.3 channels restored the fast AHP and single action potential characteristics of aged CA3 pyramidal neurons to a young-like state (Simkin et al., 2015). Notably, Kv4.2 expression is reduced in CA3 region of aged rats that successfully learned (i.e., learning-unimpaired) a hippocampus-dependent spatial water maze task (Haberman et al., 2011). Thus, we hypothesize that aged learning-unimpaired animals would have CA3 pyramidal neurons with fast AHPs that are similar to that observed in young adult animals; whereas, those aged learning-impaired animals would have CA3 pyramidal neurons with the enhanced fast AHP. Furthermore, it is possible that pharmacological intervention that restores the activity of Kv4.2/4.3 channels to young-like state in the CA3 region may also prevent and/or ameliorate the aging-related cognitive deficits, especially those related to pattern completion.

## Yin and Yang of Aging-Related Changes in the CA1-CA3 Circuit

The dichotomy of aging-related changes in the two principal neurons of the hippocampal circuit paints a major brain region trying to cope with altered local network function with progressing age. But when do these changes occur? Thus far, we have discussed the changes in CA3 and CA1 pyramidal neurons of aged animals, as compared to young adults; which are on the polar opposite spectrum of cognitive capacity. However, could these changes have started well before old age and manifestation of cognitive deficits?

**Figure 4:**
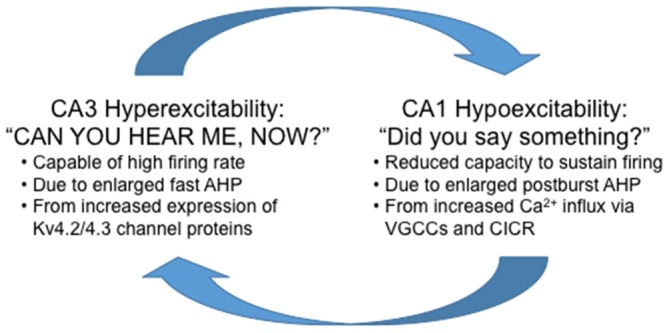
**Highly simplified schematic summary of aging-related dysfunction between the CA1 and CA3 regions.** Note that in this scheme, reducing the hyperexcitability in CA3 without increasing excitability in CA1 will not alleviate the aging-related cognitive deficits. Whereas, increasing excitability in CA1 without reducing CA3 hyperexcitability will increase information flow through the hippocampus at the expense of losing signal-to-noise and also potentially causing cellular harm from the overall hyperexcitability (e.g., seizures) of the hippocampal network.

fMRI studies suggest that hyperactivity of CA3/DG precedes changes in CA1 (Yassa et al., 2011). The increased firing rate of CA3 pyramidal neurons would send bursts of activity to its targets, the main one being CA1 pyramidal neurons. It is important to note that the medium and slow postburst AHP is observed after a burst of action potentials, hence the “postburst”, especially in CA1 pyramidal neurons. Thus, it is conceivable that with progressive increase in firing rate of CA3 pyramidal neurons with aging, CA1 pyramidal neurons would develop or use a system to limit the “extraneous” signals from CA3 to increase the signal-to-noise of information flow through the hippocampal network: or in other words, CA1 would develop a system to tune-out the extraneous noise that it is bombarded with from CA3. The most readily available system available to CA1 pyramidal neurons is to increase the postburst AHP, which would effectively increase the threshold for continuous information flow from CA3 out to the neocortical regions. Indeed, this appears to be the mechanism used with aging, as Gant and Thibault (2009) demonstrated that the enlarged postburst AHP in CA1 pyramidal neurons from aged animals effectively shunted action potentials evoked during repetitive Schaffer collateral stimulation (i.e., stimulating CA3 axons) at 7 or 15 Hz using sufficient intensity to evoke an action potential with each stimulus. Therefore, if unchecked, the natural aging-related progression of hippocampal network dysfunction is for CA3 to keep increasing its output (hyperactivity) and for CA1 to tune-out the “noise” (hypoactivity; Figure 4).

Alternatively, it maybe that CA1 became hypoactive first (due to increased postburst AHP), which led to CA3 increasing its output in an attempt to compensate. Aging-related learning studies have shown minimal learning deficit in the middle-aged animals. For example, middle-aged rats learned the spatial water maze task (Gallagher et al., 1993) and the temporal, trace eyeblink conditioning task (Knuttinen et al., 2001) as well as young adults. Yet, the postburst AHP of CA1 pyramidal neurons has been reported to be enlarged in middle-aged animals (Gant et al., 2015). Although the changes in biophysical properties (e.g., fast AHP) of CA3 pyramidal neurons in middle-aged animals are yet to be determined, it is plausible that the neural circuitry for memory formation may increase the output of CA3 pyramidal neurons to compensate for the hypoactivity of CA1 pyramidal neurons to allow successful learning to occur in the middle-aged subjects. But this again would be a temporary solution that eventually leads to a more exacerbated dysfunction of the two neighboring hippocampal regions necessary for memory formation.

## Final Thoughts and Future Direction

Regardless of the cause, the end result of the aging-related changes in CA1 and CA3 pyramidal neurons is a major disruption in communication between two subregions of a critical brain region necessary for optimal cognitive function. The ultimate questions are: can a therapeutic be developed to treat both symptoms, or will treating and/or preventing dysfunction of one subregion be enough to cure and/or prevent aging-related cognitive deficits caused by dysfunction in both regions?

Notably, calcium is the common thread that joins the dysfunction observed in CA1 (increased postburst AHP) and in CA3 (increased fast AHP). As described above, the postburst AHP in CA1 pyramidal neurons is a calcium-dependent outward potassium current (Disterhoft and Oh, 2006, 2007; Oh et al., 2013): high levels of intracellular calcium accumulation with a burst of action potentials leads to larger postburst AHP; whereas, low levels of intracellular calcium accumulation leads to smaller postburst AHP. The Kv4 mediated fast AHP also has a calcium component; as its activity is modulated by Kv channel-interacting proteins (KChIPs), which are calcium-sensing proteins (An et al., 2000; Carrasquillo and Nerbonne, 2014). Therefore, a therapeutic aimed at restoring the calcium activity and/or levels to young-like state may restore the function of these hippocampal pyramidal neurons, in addition to other cortical neurons, and prevent and/or rescue the aging-related cognitive deficits. This hypothesis is at the heart of the “Calcium hypothesis of brain aging” which is still being actively investigated today.

## Author Contributions

All authors listed, have made substantial, direct and intellectual contribution to the work, and approved it for publication.

## Conflict of Interest Statement

The authors declare that the research was conducted in the absence of any commercial or financial relationships that could be construed as a potential conflict of interest.
